# Context matters: a qualitative study of the practicalities and dilemmas of delivering integrated chronic care within primary and secondary care settings in a rural Malawian district

**DOI:** 10.1186/s12875-020-01174-1

**Published:** 2020-06-08

**Authors:** Vibian Angwenyi, Carolien Aantjes, Joske Bunders-Aelen, Bart Criel, Jeffrey V. Lazarus

**Affiliations:** 1 Faculty of Sciences, Athena Institute for Research on Innovation and Communication in Health and Life Sciences, Vrije Universiteit Amsterdam, De Boelelaan 1085, 1081 HV Amsterdam, the Netherlands; 2 Unit of Equity and Health, Department of Public Health, Institute of Tropical Medicine, Nationalestraat, 155, B-2000 Antwerp, Belgium; 3 Barcelona Institute for Global Health (ISGlobal), Hospital Clínic, University of Barcelona, Calle del Rossellón 132, ES-08036 Barcelona, Spain; 4 Health Economics and HIV/AIDS Research Division (HEARD), University of KwaZulu-Natal, Westville Campus, Private Bag X54001, Durban, 4000 South Africa

**Keywords:** HIV, Integrated care, Malawi, Non-communicable diseases, Primary healthcare, Qualitative study, Sub-Saharan Africa

## Abstract

**Background:**

With the increasing double burden of communicable and non-communicable diseases (NCDs) in sub-Saharan Africa, health systems require new approaches to organise and deliver services for patients requiring long-term care. There is increasing recognition of the need to integrate health services, with evidence supporting integration of HIV and NCD services through the reorganisation of health system inputs, across system levels. This study investigates current practices of delivering and implementing integrated care for chronically-ill patients in rural Malawi, focusing on the primary level.

**Methods:**

A qualitative study on chronic care in Phalombe district conducted between April 2016 and May 2017, with a sub-analysis performed on the data following a document analysis to understand the policy context and how integration is conceptualised in Malawi; structured observations in five of the 15 district health facilities, selected purposively to represent different levels of care (primary and secondary), and ownership (private and public). Fifteen interviews with healthcare providers and managers, purposively selected from the above facilities. Meetings with five non-governmental organisations to study their projects and support towards chronic care in Phalombe. Data were analysed using a thematic approach and managed in NVivo.

**Results:**

Our study found that, while policies supported integration of various disease-specific programmes at point of care, integration efforts on the ground were severely hampered by human and health resource challenges e.g. inadequate consultation rooms, erratic supplies especially for NCDs, and an overstretched health workforce. There were notable achievements, though most prominent at the secondary level e.g. the establishment of a combined NCD clinic, initiating NCD screening within HIV services, and initiatives for integrated information systems.

**Conclusion:**

In rural Malawi, major impediments to integrated care provision for chronically-ill patients include the frail state of primary healthcare services and sub-optimal NCD care at the lowest healthcare level. In pursuit of integrative strategies, opportunities lie in utilising and expanding community-based outreach strategies offering multi-disease screening and care with strong referral linkages; careful task delegation and role realignment among care teams supported with proper training and incentive mechanisms; and collaborative partnership between public and private sector actors to expand the resource-base and promoting cross-programme initiatives.

## Background

Healthcare systems face an increasing demand for chronic care. Non-communicable diseases (NCDs) such as cardiovascular diseases, diabetes, and cancer are among the largest contributor to global mortality, and over 70% of these deaths occurred in sub-Saharan African (sSA) and other low-income settings [1]. sSA countries continue to face a high burden of communicable diseases such as HIV and tuberculosis, which impacts individual health and the health system. This increasing double burden of chronic diseases (communicable and non-communicable) requires new approaches, including a reorientation of care that is more responsive to patients’ needs [2, 3].

Integrated care provides a possible solution to coordinate care more efficiently, especially for patients with complex needs [4]. While integration continues to gain attention in the international health discourse, its meaning and application varies widely [5–7]. First, the configuration of actors, institutions and resources in the provision of integrated care differs based on context, with different emphases placed on the pathways or forms in which integration is pursued. Second, multiple perspectives and relations among actors make it difficult to arrive at a common understanding of integration, which causes variation in defining. As such, there is limited conceptual clarity on integrated care and a plethora of terminologies used to describe integration. In attempts to simplify the concept, integration has been described as the logic of binding different health system parts together, requiring collaboration and linkages of different actors and institutions [8, 9]. Kodner and Spreeuwenberg [8] further elaborate this as “a *coherent set of methods and models on the funding, administrative, organizational, service delivery and clinical levels designed to create connectivity, alignment and collaboration within and between the cure and care sectors …*” *.* Commonly identified goals for integrated care are improving efficiency in the coordination and delivery of health services, enhancing quality of care, maximising access and coverage, and improving user satisfaction [10]. Some integration frameworks specifically addressing chronic care include the Kaiser Permanente model which stratifies the population and offers interventions based on the staging of patient’s conditions, and the Chronic Care Model focuses on reorganisation of health system inputs and processes to improve patient outcomes [9, 11]. Valentijn et al’s framework is more encompassing and is derived from the various taxonomies of integrated care reported in existing literature [4, 12]. The type, breadth, and dimension of integration varies as a result of multiple variables within and beyond a health system, for example, the set of relationships between different professionals and institutions delivering care and the characteristics of patients in need of care [9, 13].

The need to better coordinate care for patients with multiple care needs has prompted health sector reforms in high-income countries to guide the restructuring of healthcare services [11]. In sSA, health services have traditionally been structured under disease-specific, largely donor funded infectious disease programmes such as HIV/AIDS, tuberculosis, and malaria, which contributed to lowering morbidity and mortality rates [14]. However, critics argue that disease-centred models cause fragmentation of services and diversion of resources, which further weaken public health systems [13–15]. The demand for integrated care has led to a shift in practice, especially in the HIV field, which has also been instigated by an increasing number of HIV patients presenting with multimorbidities [3, 16, 17]. The evidence-base on different service models for integrating HIV and NCD programmes has grown exponentially over the years [18–24]. The scope and degree of integration identified in these models vary from integrating NCD health promotion and screening activities within HIV services, to provision of comprehensive care for HIV and NCD patients in a single location and, in some settings, the presence of explicit policies guiding integration strategies [18, 19, 21–23]. However, scaling up existing integrated care initiatives has proved challenging, since these are pursued within contexts facing multiple impediments, including health workforce shortages and inadequate health financing [22, 25].

Malawi experiences a high HIV burden, with 1 million people are living with HIV [26]. NCD incidences are rapidly increasing, whereas in a 2009 nationwide STEPS survey of 5206 adults, 30% had hypertension and 8.9% reported a cardiovascular disease [27], recent statistics estimate the probability of death from the top four NCDs is 16.4% [28]. Tackling these problems has prompted the country to institute several reform initiatives. For HIV care, reforms include decentralising antiretroviral therapy (ART) to the community and primary healthcare level, and strengthening community-based structures to track and trace patients lost to care [29–31]. Other innovations include integrating HIV care with other services; such as tuberculosis screening and treatment, reproductive health, cervical cancer screening and, more recently, hypertension screening was included in the 2016 HIV guidelines [32]. Prior to the development and implementation of national NCD policies since 2011, isolated efforts to integrate NCD care within HIV services existed [32, 33]. For instance, one southern district, established an integrated chronic care clinic which enabled patients to access a continuum of care in a single visit [34]. However, significant gaps remain in the provision of universal and comprehensive health services to patients with other chronic conditions beyond HIV at the primary level. Recognition of and strategies for addressing these gaps have been articulated in the country’s current health strategy and include a focus on integrated care [35].

Since the evidence-base on integrated care largely comes from Western settings, more research is needed to identify strategies and forms of integration in resource-constrained sSA health systems, to expand our understanding of integrated care within these contexts. Particularly pertinent is the focus on integrated care application at the primary level, where the majority of people in sSA access their health care. Integrated care emerged as an important theme in our study on chronic care in a rural Malawian district, and in this paper, we explore the current practice of delivering integrated care for chronically-ill patients with a particular focus on the primary level, the (potential) challenges to integrating care, and implications on practice and response measures. We present our findings using the integrated care framework developed by Valentijn et al. [4]; a framework informed by an extensive review on integrated care conceptualisation and deemed suitable for our particular study as it takes the primary care level as its point of departure for integration across the care continuum.

## Methods

### Study design

This paper is based on a sub-analysis of qualitative data from a larger mixed methods study, which examined local models for chronic care and self-management support in southern Malawi [36]. The primary dataset was collected within two rounds (April to July 2016, and March to May 2017), in Malawi, involving multiple methods and respondents [37]. The data presented in this paper focuses on the concept of integrated care and how it is shaped and applied at the lower level of care. Our sub-analysis was guided by the following question: how and to what extent are integrated care approaches utilised in local models for chronic care?

### Study context

This study was conducted in Phalombe, a rural district located south-east of Malawi near the border with Mozambique with a population estimate of 390,000 [38]. The district has the lowest socio-economic indicators compared to the other 27 Malawian districts. In addition to infrastructural challenges, it has high illiteracy and poverty levels, with the majority of residents depending on farming and small-scale trading [38]. Healthcare in public facilities is universally free at the point of care, while services offered in private facilities, mostly operated by the Christian Health Association of Malawi (CHAM), charge user fees. The majority of residents access care in public primary healthcare (PHC) facilities, which consist of health centres, dispensaries, and health posts [35]. Given the absence of a government-owned hospital, secondary care is provided in a district public health centre, although most referrals from PHC facilities are directed to the district mission hospital [38].

### Data sources, sampling, and data collection procedures

A document analysis was conducted with the following purpose; to interpret the policy context on chronic care delivery and how integrated care is conceptualised and instructed at the service level. VA conducted the search of related documents and identified the information for assessment. Document sources included district implementation plans and health facility records, clinical guidelines for HIV and NCDs, and different policy documents such as the National Health Sector Strategic Plan [35, 39, 40].

#### Key-informant interviews

Fifteen interviewees were selected, using the purposive sampling technique, in consultation with senior health managers and health facility administration. Interviewees were identified from five of the 15 district health facilities, which represented the district health services structure and various levels of care (i.e. three primary level facilities and the only two secondary level facilities providing referral services). These facilities were part of the catchment area of our larger study [36]. Interviewee selection aimed for representation and diversity by gender, professional cadre (clinical, non-clinical, and health managers with varying responsibilities in chronic care), and health facility category (public and private; PHC and referral facilities).

Topic guides for the larger study were developed based on themes identified from the literature on chronic care and from themes emerging during data collection (see File S1). This paper presents a sub-analysis of the primary dataset, in which integrated care was explored in more depth, following an abstraction of our findings around: 1) perceptions of the organisation of chronic care services and local challenges in implementing national strategies and guidelines such as those for NCDs and HIV; 2) innovations in service delivery and opportunities for cross-learning across different disease-specific programmes; 3) collaboration between public and private sector actors and its impact on integration; and 4) potential challenges to integrating care including health services financing. The topic guides were pre-tested at first use and some minor, mostly language, changes were made.

Interviews were held in private rooms/interviewees workstation, lasting between 45 and 90 min, at a convenient time in accordance with pre-booked appointments. Interviews were conducted by VA, a female doctoral research fellow with extensive qualitative research experience, with support from a trained female Malawian research assistant trained on qualitative research methods. The interviews were mostly conducted in English except two, which were held in the local language *Chichewa,* then later translated to English. Audio files were transcribed verbatim by the research assistant and verified by VA. Personal identifiers were replaced with codes for anonymity. In File S2, we reflect on our positionality in the research process and report this using the Consolidated Criteria for Reporting Qualitative Studies (COREQ) checklist.

#### Structured observations and meetings

Structured observations in the five health facilities studied aimed at understanding and documenting how chronic care services were organised in the district. Observations conducted by VA and the research assistant at PHC level clinics covered HIV and general outpatient services, and at district referral facilities, palliative, NCD, and mental healthcare services. During these single facility visits, information documented included the type of services provided, personnel responsible, client flow, and approaches in service delivery. A structured observation tool developed for this study was used to guide notetaking and in drafting the final comprehensive report (see File S3). Data on patients’ experiences with health services and interaction with service providers in the health facilities included in this study are reported in separate publications [41, 42]. We visited five non-governmental organisations (NGOs) implementing donor-funded projects in the district to study their programme activities, and understand their working relations with the district health office in supporting chronic care services. These organisations were identified through discussions and referrals from key-informant interviewees. The focus of each organisation varied: three organisations focused on various aspects of HIV care, one offered community outreach services, and one focused on health system strengthening. Discussions with representatives from each NGO, i.e. senior project staff, were open-ended lasting 30 to 60 min, guided by the above broad topics and captured in detailed hand written notes.

### Data analysis

Data from all sources (interview transcripts, structured observations, meeting reports and data extracted from document analysis) were managed using NVivo 11 (QSR international) and analysed with a thematic analysis approach [43]. The coding framework, which included codes emerging from the data and deductively from the topic guide questions was developed by VA in consultation with CA. VA read the transcripts, coded the data and generated charts of grouped themes, which were exported to Microsoft Word to explore emerging patterns and shared with co-authors for further interpretation. For our sub-analysis, we used the charts to discuss and compare between the grouped themes and patterns with a view to further articulate the major categories (informed by both Valentijn et al’s integrated framework and the data itself). These formed the basis for organizing and presenting the research findings. At the end of the larger study, representatives from the participating health facilities and district health management team attended feedback meetings (*n* = 5), where findings were shared.

### Ethical considerations

Ethical approval was obtained from two institutions (EMGO+ WC2015–080, 27-Oct-2015; NCRSH P.11/15/64, 10-Dec-2015). At the point of introducing the study and obtaining informed consent from research participants, the interest in the research topic, which formed part of the first author’s doctoral thesis was openly declared. All eligible participants approached for interviews provided written informed consent, and verbal informed consent was obtained to conduct observations and meetings related to the study.

## Results

Table 1 below presents characteristics of study participants. The results are discussed under four thematic areas which relate to the integration concepts as described by Valentijn et al. [4]. The areas include: system integration, which examines the current policy framework and how integration is perceived; organisational integration of chronic care interventions and efforts towards implementing an integrated approach; health workforce capacity for professional integration; and factors potentially affecting integration efforts (e.g. health financing).

**Table 1:** Sampling and participants characteristics

**Category**	**Health worker cadre**	**Gender**
District health managers	District health officer District nursing officer HIV programme coordinator NCD programme coordinator Palliative care coordinator District AIDS coordinator	4 males 2 females
District private hospital	Hospital matron Health information officer Chief clinical officer	2 males 1 female
Public health centre	Nurse Health surveillance assistant (HSA)	1 male 1 female
Faith-based health centre	Nurses Chief clinical officer Health surveillance assistant (HSA)	2 males 2 females

### The policy framework and how integration is perceived and practiced

A prerequisite to facilitate system integration is the presence of a supportive policy framework. The concept of integration has been mentioned in a number of Malawian policy documents. For instance, the National Health Sector Strategic Plan II (2017–2022), which provides an overarching national strategy for implementing health services, emphasizes ‘efficiency and effectiveness’ as important guiding principles [35]. The strategy states: “*… opportunities shall be created to facilitate integration of health service delivery to leverage on efficiency and effectiveness in addressing health needs of people …*” [35]. Through promoting coordination and collaboration between actors operating in health and other sectors, the strategy envisions reduced fragmentation and duplication. Furthermore, in delivering chronic care, specific interventions are outlined in the national NCD action plan and HIV clinical guidelines [39, 40]. The NCD action plan frames the interventions within a multidisciplinary and collaborative approach to promote the delivery of a continuum of care in a holistic and integrated manner [39]. The 2016 HIV guidelines, emphasise on the delivery of interventions at various service delivery points such as different departments within a health facility (horizontal integration) and across different levels of care (vertical integration) [40].

Some of the programme coordinators interviewed spoke of the importance of coordinating the programmes and activities, which target chronically-ill patients (both those with HIV and an NCD). They reported having focal persons at the district level for HIV, NCD, palliative services, and mental health. This was aimed at facilitating coordination of chronic care interventions within facility and community settings, and delivered by Ministry of Health (MoH) personnel and district partner NGOs. Although health interventions have been organised under distinct vertical programmes as stipulated in Malawi’s essential health package [38], the coordinators interviewed reported that they had a core responsibility to pursue opportunities for linkages across different programmes and levels of care. These attempts were made through joint planning review meetings organised at the district health management team level, and in liaison with NGO representatives. However, this largely depended on the nature of relations, reporting and communication systems, in addition to the extent of collaboration forged by these set of actors.

*… sometimes there are partners [NGOs] who we [MoH] sit down and discuss what the partners have, and we tell them what we want … or what we are looking for, and what they are doing may not match, but because they have drugs, we can discuss with them and they help us.****(KII15_Manager_District)***

Coordination of different professionals and health partners as a key mandate of the district authorities was identified needing strengthening as interviewees suggested the need for joint planning meetings especially in the formulation of district implementation plans, and increasing participation in district stakeholder meetings where all health and non-health sector stakeholders are engaged, usually organised by the District Commissioner or using other networking forums (see File S4, quotes 1–3).

### Organisation of chronic care and efforts towards implementing an integrated approach

Within PHC settings, our observations revealed variations in the delivery of chronic care as summarised in Table 2. In most of the PHC facilities, NCD services were provided as part of general outpatient services. For HIV, efforts were made to integrate HIV services across different service points within private and public facilities including within maternal and child health clinics, general outpatient, and youth-friendly service stations. This differentiated approach to HIV service delivery, it aimed at catering to the specific needs of a diverse patient population such as lactating mothers with their HIV exposed infants, and adolescents living with HIV. Furthermore, HIV services integrated testing and screening for tuberculosis, cervical cancer, and other opportunistic infections at the point of care. To strengthen referral linkages and close patient monitoring, health facilities worked closely with HIV expert-patients and community-based structures operated by community health volunteers, with the latter providing a range of health and social services (see File S4, quotes 4–5).

**Table 2:** Organisation of care for selected chronic conditions at primary and secondary level

**Category**	**HIV care**	**Diabetes and cardiovascular conditions**	**Mental Health (Epilepsy)**	**Palliative care (Cancer)**
*Organization of services and level of care*	Stand-alone clinics on designated days of a week; special clinics for different groups e.g. pregnant and lactating mothers, teens and young adults	Consultation visits in outpatient clinics of PHC facilities; joint diabetes and hypertension clinic in district health centre	Consultation visits in outpatient clinics of PHC facilities; Selected clinic days for mental health and palliative services in the district health centre	
*Medical supply*	Antiretroviral treatment constantly available in all facilities	Most drugs in the essential medicines list are frequently out of stock; patients referred to paying or secondary level facilities		Essential cancer treatment and palliative care provided in 3 health facilities
*Screening and diagnostic resources*	Vital measurements taken as per treatment guidelines; screening for tuberculosis and cervical cancer screening (VIA) available; results of HIV viral load sometimes delay	Resources for screening and diagnostic limited in public PHC facilities; patients referred to private PHC or referral facilities		
*Cost and affordability of services*	Free HIV services in all health facilities (public and private). Includes consultation, diagnostics, and treatment	Free health care in all public health facilities. Includes consultation, diagnostics, and treatment. Out-of-pocket payment when patients referred to private facilities		
*Health workforce*	Multi-disciplinary team with designated tasks i.e. clinicians, lab technicians, data clerks, health surveillance assistants, and HIV expert-patients	Clinician on duty provides consultation services (inadequate numbers and skills-mix); health surveillance assistants support health education, triage, and documentation		
*Health information system*	Most public health facilities use electronic medical record system. Patient information recorded in health passports	Documentation is mostly paper-based records; patient information recorded in health passports		
*Other initiatives*	Differentiated care: Preventing mother to child transmission (OPTION B+ approach); HIV teen and youth clubs; HIV discordant couple programme Community support: self-formed patient support groups; home-based care through community health volunteers HIV expert patients support HIV care in clinics and patient homes	Community support: home-based care through community health volunteers NCD master card; data reporting tool for hypertension and diabetes introduced at the district health centre	Community support: home-based care through community health volunteers	

*Acronym: HIV* human immunodeficiency virus; *NCD* non-communicable diseases; *PHC* primary health care; *VIA* visual inspection with acetic acid

*Notes:* OPTION B+ is a prevention of vertical transmission approach to HIV positive expectant mothers and treatment initiated immediately, and continued for life

At community level, outreach clinics were organised to target hard-to-reach communities, offering care closer to patients and facilitating vertical integration. One NGO offered health promotion and curative services, including for some chronic conditions, during weekly mobile clinics. The district health office dispatched a team of clinicians to all its PHC facilities during scheduled clinical outreach visits, offering both general and specialist care. However, the regularity of these clinics depended on availability of transport, materials and on the proper publicising of these events to community members, as one respondent explains (see also file S4, quote 6):

*… like NCDs and mental health, we do outreach clinics like every Thursday, so we move around health centres. If we are provided transport, and have enough drugs, and the staff are enough, we go around because it is like a team. We go with the orthopaedic, ophthalmologist, general clinician, psychiatric nurse, pharmacy and even laboratory [staff] …****(KII15_Manager_District)***

At the secondary/referral level, the district health centre (equivalent to a level II health facility) initiated a clinic for patients with diabetes and other cardiovascular conditions, operating twice weekly with NGO support. Palliative services were initially accessible only at secondary level, but since 2017, two public PHC facilities were elevated to offer these services to cancer patients, improving access in remote settings.

Interviewees reported several factors hampering the extent of integration in delivering chronic care, especially in public PHC settings. One barrier was the limited physical space. Due to the sheer volume of HIV patients and limited consultation rooms in public health facilities, HIV clinics operated as stand-alone clinics on designated days of the week, to address challenges encountered. Consequently, this implied marshalling a substantial portion of health facility staff to run these special clinics, which restricted operation of other clinical services. One manager described this challenging situation as:

*R: … we lack enough consultation [rooms]. The same consultation where the NCD clinics is happening … the [following] day is another clinic … if we had a little bit considerable number of consultation [rooms], then anybody can come and be provided with services …****(KII19_Manager_District)***

Other barriers interviewees reported were linked to the non-availability of medical supplies and health service infrastructure. Most facilities lacked basic screening equipment such as blood pressure monitors, glucometers, and urine dipsticks to enable point-of-care diagnosis for NCD conditions. Hence, patients were often advised to seek these services in private PHC or referral facilities at their expense. The absence of a central HIV diagnostic and laboratory unit meant transferring samples to other districts, affecting timely analysis. A notable challenge for most public PHC facilities was the interrupted supply of essential NCD drugs, including for diabetes and epilepsy reported to last for several months, which affected availability of comprehensive services for this group of patients (see file S4, quotes 7–9).

*… last time we had no Glibenclamide for diabetes and currently we do not have … Metformine, which means [patients] are suffering … like epilepsy, we have one drug and before five drugs were out of stock … if the Central Medical Stores [National level] does not have the drugs, we [district health team] do not have the capacity to procure …****(KII15_Manager_District)***

### The health workforce capacity for professional integration

Many districts in Malawi, including Phalombe, experience severe health workforce shortages. Only 55.1% (*n* = 901) of Phalombe’s health workforce positions are filled, with a critical shortage of clinical staff (Table 3). Observations made at the different facilities showed disparities in the distribution of staff, both in volume and cadre. HIV programmes were executed by multidisciplinary teams consisting of trained cascade monitors, triage staff, health promotors, defaulter tracers, data clerks, and others (see Table 2), while palliative, and mental health programmes had more limited team compositions, and lacked a base of trained providers.

*R: … we do not have personnel who can do clinics for other chronic conditions … it will be good if the Ministry or stakeholders train a few individuals who can [operate] these clinics, because if people [patients] are always told to travel to Phalombe [district referral] … they could access the same services here [PHC facility] … (****KII09_Nurse_Private-PHC)***

**Table 3:** District health workforce both public and private health facilities^a^

**Cadre**	**Required position**	**Current staff total**	**Percentage filled position**
***Clinical/facility-based practitioners***			
Medical officer/specialist	11	2	18.2%
Dentist	1	0	0%
Pharmacist	2	0	0%
Clinical officer/ technician	113	20	17.7%
Pharmacy technician	20	5	25%
Nursing officer registered nurse	38	21	55.3%
Nurse midwife technician	322	69	21.4%
Medical assistant	51	23	45.1%
Mental health staff - clinical	0	0	None
Mental health staff - nursing	2	0	0%
Community midwife assistant	6	12	200%
Pharmacy assistant	10	2	20%
Lab officer	1	0	0%
Lab technician	21	6	28.6%
Lab assistant	15	1	6.7%
Dental therapist	17	3	17.6%
Radiographer	3	2	66.7%
Radiographer technician	4	2	50%
Home craft worker	48	13	27.1%
***Public/community health practitioners***			
Nutrition staff	1	0	0%
Education/Environmental health officer	24	9	37.5%
Health surveillance assistants	250	217	86.8%
***Other category***			
Missing	0	1	Not specified
Not included	676	445	65.8%
**Total**	**1636**	**901**	**55.1%**

^a^*SOURCE:Human Resources for Health audit for year 2016/2017 obtained from Phalombe District Health Office*

The few clinicians offering NCD services at general outpatient departments reported lacking technical knowledge and expressed a desire for further training. The limited availability of key cadres such as community health nurses and public health officers, principally trained to coordinate community-based services like health promotion and outreach activities was another reported challenge. This resulted in the unstructured delegation of responsibilities to Health Surveillance Assistants (i.e. government-paid community health worker cadre in Malawi), who were overstretched with other programme activities and lacked proper training in the management of chronic conditions.

The necessary components for integrated care such as the provision of supportive resources, capacity-building opportunities, and a work environment that facilitates an integrationist approach were explored among health managers interviewed and in meetings with NGO representatives, who described the approaches and challenges of putting these components in place (see File S4, quotes 10–11). First, the need to avail clinical guidelines/job aides within workstations was recognised, although access to NCD supportive resources was limited, and mainly attributed to the nascent NCD programme at national level. Second, mentorship and training opportunities were largely programme-led and depended on resources allocated to support for instance senior staff in conducting regular supervision visits in PHC facilities. Initiatives introduced by NGOs working in collaboration with the district health office, such as having an integrated information system (NCD master cards and electronic medical records for HIV patients), were perceived by some of the respondents to improve documentation, enhance monitoring and evaluation, and improve patient management. In addressing the health workforce shortage, NGO project staff were entrenched in public health facilities and worked alongside existing MoH personnel to support with clinical care. Furthermore, the liaison between MoH frontline providers and volunteer-operated community-based structures was also seen as an opportunity to assist healthcare teams to fulfil the role of a more community-oriented primary care team.

### Issues of financing

Malawi’s health sector is largely externally funded (61.6% donor funds) and funding allocation not equally matched across various programmes; for instance, of the USD 663 million total expenditure on health in the 2015/2016 financial year, over 30% was allocated to HIV compared to less than 10% to NCDs [44]. The national government, through the fiscal transfer framework, disburses revenue to district authorities to implement approved budgets linked to the district implementation plans. Health managers interviewed reported that their district implementation plans were essentially informed by a resource mapping exercise of programmes implemented by NGOs, followed by an allocation of funds to under-resourced programmes through the sector-wide approach. For instance, given that the majority of NGOs present in the district supported the implementation of HIV services, there were attempts to ensure health budget funds were prioritised and directed towards NCD and mental health activities. Thus, the financing of a complete service package at different service levels in the district depended largely on the flexibility of NGOs to also accommodate non-programme activities (see file S4, quote 12). This dependency affected both the autonomy and ability of district managers in setting out their course under guidance of national level strategies:

*… because we are resource constrained and manpower constrained, we [district health department] are not on top … these [NGOs] come in the district and then we recommend to them over and above what they have designed for their services …****(KII18_Manager_District)***

Over one-third of health services in Malawi are offered by private-sector providers, and predominantly under the Christian Health Association of Malawi (CHAM) [35]. The national government sub-contracts CHAM health facilities to provide subsidized healthcare. Interviewees reported that patients incur costs for services not included in the service package, especially for NCDs and expressed the need to support NCD care (see file S4, quote 13). This acts as a deterrence to access and affordability of pro-longed care as expressed by the following respondent:

*… they [patients] pay consultation fees and they have to pay for drugs and lab tests … but because it is a life-long problem for them [patients] … some of them are being assisted by their children since they are no longer productive … It [healthcare services] should be sort of like universal, irrespective of where they go; whether to a private hospital or government hospital they [patients] should get free services.***(*KII06_Clinican_District)***

As a border district, the national ‘free’ public healthcare policy attracts patients from neighbouring country to Phalombe, which results in overstretched services within some of the public PHC facilities. Porous borders and challenges of vetting non-resident patients at the point of care puts additional pressure on district health services and budget.

… *[some] of the people that access this health facility are from Mozambique … If those people were paying something, at least we might have some resources, or we could add more resources to the already few resources that we have.****(KII03_Nurse_Public-PHC)***

## Discussion

Our study provides qualitative evidence of the practices and complexities of optimally employing an integrated approach in delivering chronic care for HIV and NCD patients at the primary care level in a rural Malawian district. Our results show how the continued, vertical organisation of disease-specific programmes, policies, and financial allocation affected this approach. The rationale for disease prioritisation as well as the implications of such approach for African health systems have been widely documented [4, 13, 45–47]. This study adds to this body of knowledge by highlighting the current disparities between HIV patients and those with NCDs in terms of their access to essential health services at the primary level. For both patient groups, provision of an integrated care package is severely hampered by the frail state of primary healthcare and the still nascent presence of NCD-specific services. Similar situations are observed in other Malawian districts, with a recent report revealing that approximately 10–20% of all health centres are fully equipped to treat common NCDs [48].

The ongoing experimentation with integrated care in Malawi and surrounding countries in the region have produced important lessons on how to pursue and strengthen integrative strategies at the primary care level. With regards to system integration, our results indicate that integration is mentioned in health policy documents, however, proper implementation of integration policies requires coordination of programmes and activities, improved stakeholders collaboration and combination of both horizontal and vertical integration approaches. However enforcing system changes is a challenge in most resource constrained contexts. For instance, while the ideal is to have multi-purpose frontline health facilities, where patients’ access a continuum of services irrespective of their conditions, our results bring to the forefront challenges in the availability and organisation of resources to support integration. Studies on integrated HIV-NCD care demonstrate that improving patient flow processes such as sorting appointments and referring patients to the appropriate service stations (e.g. for drug refill or consultation with a clinician) could be beneficial to facilities experiencing high-volume patient load and operating within limited space [22, 24, 34, 49]. As part of primary prevention strategies and policy recommendations, including multi-disease screening within triage processes promotes early detection and management of complications associated with chronic diseases, though it requires ensuring diagnostics are regularly available [23, 50]. Our results show clear deficiencies in the availability and capacity of the health workforce, further complicated by sub-optimal working conditions to offer integrated care. Tackling health workforce deficiencies requires enforcing carefully crafted task-shifting strategies to redistribute tasks and redefine healthcare teams’ responsibilities, especially where teams have to embrace ‘newer’ responsibilities and include lay health worker cadre [25, 51, 52]. In our study context, a further engagement of state-paid health surveillance assistants in absorbing tasks such as patient education, triage, and documentation could help ease clinician’s workload and allow them to focus on technical tasks. Complementary to these efforts, requires ensuring the requisite clinical resources are available, offering regular training and supportive supervision to health professionals and other personnel offering community-based services [49, 52]. The gaps in service provision at facility level that were reported could be mitigated by expanding service points at community level. In rural underserved settings like ours, community-outreach clinics can be utilised to offer multi-disease screening services, treatment and early referrals, as demonstrated in Uganda [53].

The initiation and scaling-up of integrated care strategies implies increased costs, though this should result in more efficient services, improved health outcomes, and cost-savings over time [9]. As our findings suggest, the prevailing health-financing situation characterised by lean national funding for some chronic conditions, over-reliance on donor-funded projects, and inadequate domestic financing puts immense pressure on efforts towards integrated care provision [32]. In tackling these challenges, we acknowledge it requires collective efforts by all actors including national government and private sector/donors, and more importantly thinking beyond investment for disease-specific programmes to focus more on strengthening health systems. On this basis, we make the following proposals. At national level, expansion of health budgets, provision of technical support to sub-national structures, and redirecting developmental aid towards sector-wide initiatives are worth. At district level, more efforts are needed to facilitate cross-programme coordination and investment channelled to improve primary care operations, including strengthening medical supply chain and infrastructural upgrades. The prominent role of private-sector providers in Malawi’s healthcare system prompts the need for national government to review and negotiate service level agreements, to facilitate expansion of essential health service packages that accommodate provision of critical services, especially for NCDs. Through this, the promise of achieving universal health coverage by providing free to subsidized chronic care services at primary level addresses the financial barriers to accessing care Malawians face, particularly those residing in rural settings [54].

### Limitations

This study was conducted in a single rural Malawian district, hence generalizability of findings is limited. Our study synthesized findings from healthcare providers and health managers interviews, structured observation of healthcare practices to understand features of integration in this context. However, there are important factors beyond what we studied such as patient systems, cultures and norms which influence integration practices [13]. Our study did not explore to what extent integration practices impact on outcome measures such as organisational efficiencies, which may require applying other methodological approaches such as clinical audits or review of programming documentation/data. Using Valentijn’s framework [4], was helpful in conceptualising integration in the primary care context, although our data was limited in exploring all dimensions of integration equally. Future research could focus on assessing how integrated care impacts on patient outcomes especially those with multimorbidities, and its relevance in health system strengthening initiatives especially in low-income countries.

## Conclusion

The provision of integrated care for patients requiring long-term care has become an important health system reform, and there is growing evidence on how these reforms unfold in practice. Our findings point to the complexity of implementing integrative strategies within resource-limited PHC settings like rural Malawi, and to the progress made in the face of such complexities. Contextual factors, such as staffing levels and an infrastructure of vertical disease programmes influenced the practical implementation of national policies and strategies in support of integrated care provision. Further investment and support is needed towards primary healthcare provision in general and NCD services, particularly in terms of clinical resources, ensuring regular supplies, and provision of training and mentorship to frontline providers to enable them offer integrated care as close to the community as possible.

## Supplementary information

**Additional file 1.** Interview topic guides.

**Additional file 2.** COREQ checklist.

**Additional file 3.** Health facility structured observation guide.

**Additional file 4.** Additional quotes.

## Data Availability

Freely available data includes data in results table, supplementary files, in summary form under main headings, and anonymised quotations from interviews. Access restrictions applies to some data, such as interview transcripts, due to potentially sensitive information and may contain identifiable information. Hence sharing of original transcripts may be a threat to confidentiality and breach of data sharing agreements included in the consent forms used in this study. Additional approvals will be required from the ethical review committees for applications to re-use data, in line with informed consent agreements. Applications can be submitted to the National Committee on Research in the Social Sciences and Humanities, Malawi (ncrsh@ncst.mw); and the EMGO+, Vrije Universiteit Amsterdam, the Netherlands (wc.emgo@vumc.nl).

## Abbreviations

ART: Antiretroviral therapy
CHAM: Christian Health Association of Malawi
CHBC: Community home-based care
HIV: Human immunodeficiency virus
HSA: Health surveillance assistant
MoH: Ministry of health
NCD: Non-communicable disease
NGO: Non-governmental organisation
PHC: Primary healthcare
sSA: Sub-Saharan Africa
VIA: Visual inspection with acetic acid
