# Supplementary material for: Context matters: a qualitative study of the practicalities and dilemmas of delivering integrated chronic care within primary and secondary care settings in a rural Malawian district
Source: BMC Fam Pract. 2020 Jun 8;21:101. doi: 10.1186/s12875-020-01174-1 (PMC7282183; doi:10.1186/s12875-020-01174-1)
Supplement: Supplementary file 1 — Additional file 1. Interview topic guides. [file 12875_2020_1174_MOESM1_ESM.docx]

## Topic guide 1: Senior health officials interview guide

**RESPONDENT DETAILS**

*Names (optional)*

*Gender:*

*Designation:*

*Venue:*

*Date:*

*Start time:*

*End time:*

**INTRODUCTIONS**

Introduce self and accompanying colleague. Revisit objectives for the discussion: to learn from senior official their role and office in supporting delivery of chronic care, learn of recent developments and the strategy and policies guiding implementation. Provide information sheet, seek consent for recording and explain purpose for doing so.

To start us off, I would like to know as ….what are your day to day responsibilities as the office holder?

- For how long have you been working in the district and where were you before/previous posts

**DHO/DHMT REPRESENTATIVES**

POLICIES AND STRATEGIES

When we look at the delivery of chronic care services at the district, what are the national strategy/policy documents in use to guide implementation e.g. HIV, what about NCD and mental health, what about CHBC? ***(Request for copies if available at the end)***

- Do these policies address the local disease burden and whether it is feasible to implement them accordingly
- What is most challenging for this district? *e.g. relatively new district, limited infrastructure, resources and population coverage*

FINANCING AND RESOURCE SUPPORT

- Could you comment on the district health budget in comparison to the national health budget? How much is allocated and how is this distributed e.g. chronic care vs general services
  - Are there any gaps and how do you deal with funding challenges?
- How do health partners/other sectors contribute in terms of financial and other resource support. *Probe for examples (role of CHAM, NGOs, DC and external funding/private donors)*
- Could you comment on the free health care (UHC) policy, especially looking at chronic conditions:
  - What are the challenges in implementing this policy?
  - Feasibility/current thinking to finance chronic care in the long-term with a growing burden of HIV and NCDs?

HUMAN RESOURCES

Let’s look at general staffing and health facilities at the district:

- Currently how many health facilities are registered under the DHO: CHAM/PHC, health posts etc.
- Let’s talk about health workforce, what is the % of posts filled in the district e.g. no. of doctors, nurses, community health nurses, medical assistants, data clerks, HSAs etc
  - Could you comment on general health worker distribution e.g. in primary health care facilities. *Prompt level of qualification, gender and numbers.* ***(Request at the end to get actual figures from human resource/administrator)***
  - Do you experience any particular challenges *with* health worker distribution in the district health facilities *e.g. in distant facilities, staff turnover (study leave/new positions)?*
  - Is there a strategy in place to address this challenge e.g. working with partners *Let’s talk about non-professional health workers (e.g. Expert clients, volunteers etc), in your opinion what is their added value and what seems to be a good skill/provider mix to render chronic services.*
- In terms of capacity building, how are health workers supported with on-going training to improve service delivery for chronic care services?
  - State of supportive supervision?
  - Who are targeted with training? i.e. coordinators, clinicians, PHC staff?
  - Is it same/different for certain diseases/departments e.g. HIV, NCD, mental and palliative care?
  - An observation in the previous round was the gap in training for NCD and mental health care, especially for PHC staff and lay health workers (plus HSAs) were not well prepared with knowledge/training? Could you comment on that?
  - In terms of patient self-management education; at provider level how can this be promoted at health facility level? (i.e. moving beyond general health education and empowering patients with skills/information)
  - What is being done to address this gap?

HEALTH SERVICE ORGANISATION AND DELIVERY CAPACITY (CROSS-CUTTING ISSUE)

Let’s now look at the different departments related to chronic care: If possible, ask the following questions for each department

*1. HIV services*

*2. NCD services*

*3. Mental health services*

*4. Palliative care services*

- For instance in terms of available recourses i.e. guidelines/job aides, drugs availability, diagnostics, reporting tools?
- What are current/ongoing initiatives to improve these services at the district (are there partners involved)
- What about the capacity to deliver these services at PHC;
  - what seems to be working well
  - What seems to be lacking
- Besides health facility care, we observed there are outreach clinics for (general and specialised care) and community and home based care provided by volunteers and family caregivers.
  - An observation was the rota for monthly PHC clinic camps. Do they still happen and how frequently?
  - How are they working with NGOs/health partners to expand services at community level?
  - *How are community-based groups being supported and engaged with the DHO/health facilities? Is there scope for improvement (how)?*
    - CBOs/FBOs
    - Patient support groups

CHRONIC CARE MODELS

- As we wrap up, looking at how chronic care services are organised at the district level, what are your proud achievements/what seems to be working well?
- What are areas that are still of concern? E.g. from our observation that these are still stand-alone services HIV and NCD care; patient focus vs promotive population-based activities
- How do you envision dealing with these issues

## Topic guide 2: Health workers interview guide

**INTERVIEW DESCRIPTOR DETAILS** *(Confirm at end of interview all details are captured – separate sheet)*

1. **INTRODUCTION**

To start us off, I would like to know more about your roles working in this health facility:

- For how long have you been working in this facility?
- Where were you previously and what were your roles?
- What is your training and background?
- What are your main roles in this facility?
- In the community, what are the activities you conduct in relation to your job? *[especially HSAs, community nurse, expert client]*
  - *Probe: identification/tracing of patients at risk; HTC; home visits; health education; counselling (ask what topics); referrals from communities/home to health facilities and follow-up/monitoring*
- Let’s now focus on patients with chronic conditions (mention examples), what are your main roles/responsibilities in terms of providing services?

1. **PERCEPTIONS OF HEALTH CARE AT THE HEALTH FACILITY**

Let’s now focus more on chronic conditions:

- What are the common chronic conditions you deal with at the facility and why is that the case?

What are the general perceptions in the community about the services offered at this health facility for patients with chronic conditions (mention examples)?

- If ok, what is liked? *(follow-up with probes)*
- If not ok, what are the concerns? *(follow-up with probes)*
- Are there patients with chronic conditions in the community who are not accessing health services and why is that the case? *(ask them for specific examples)*
- Where else do they go to seek health care services *(e.g. nearby health centers, practice the use of alternative medicines)* and why is that the case?
- As a faith based OR government health facility *[select appropriate choice]* any particular challenges faced with delivering services for patients with chronic conditions? (ask for details)

1. **SELF-MANAGEMENT SUPPORT IN CHRONIC CARE**

**FOR CLINICIANS ONLY:** Let’s now talk about patient self-management for chronic conditions

- In your opinion, how would you describe self-management/self-care when referring to patients with chronic conditions [give example]?
- How can patients with chronic conditions be supported to take care of themselves (ask for details)

**FOR ALL:** Let’s look at patients with chronic conditions and their needs

- What do you consider as important needs for such patients to help manage their condition *(e.g. proper diet, shelter, financial support, medication, psycho-social and spiritual support, access to various health services)*
- Are there any difficulties meeting these needs and why is that the case?

**FOR ALL:** Let’s talk about patient adherence to treatment:

- Do you have patients with problems adhering to their treatment? And to what extent is this a problem?
- What are the reasons for non-adherence to drugs/medication and clinical advise?
- Is this a problem for patients with certain conditions *[e.g. HIV vs other chronic conditions – give example]* or for patients with particular characteristics *[e.g. age, gender, literacy levels, residence, religion]* (ask for specific examples)
- What measures have been taken to encourage patients to adhere to their treatment?

**FOR ALL:**

- How is the health-facility and personnel equipped to provide services for patients presenting with different chronic conditions **(what is working or not working well). I will now discuss with you specific issues of interest. For instance:**
- Availability of medical technologies: *Probe: drugs, diagnostic equipment, specialized tests e.g. Diabetics, BP, Epilepsy*
- Service delivery: *Probe: extent of focus on health promotion/preventive vs curative; general vs special clinics for certain conditions; facility based vs community based activities.*
- Staffing level: *Probe: adequacy in numbers, responsibilities and delegation of tasks [by department], cadre/qualification and what is lacking (community health nurses)?*
- Level of skills and training of health personnel (both professional and lay volunteers):

*Probe: type of training and who gets to attend e.g. home based care, palliative care, HTC, ART, other chronic NCDs etc.*

- Use of guidelines and treatment protocols: *Probe: whether they are referring to any job aides/guidelines for their work e.g. HTC, home based care, ARV etc.*
- Financing of health services: *Probe what services are free or need payment*
- Referral system *Probe: tracing and linking patients from homes/communities to local health facility (community referral); facility to facility based referrals; ambulance services*
- Health records: *Probe:* *paper based vs electronic based, integrating records for patients with multiple conditions [HIV+]*
- Communication and productive interaction with patients *Probe: encouraging patients to ask questions; providing counselling; providing adequate information; linking with other support network.*
- I would like to understand what MAJOR challenges you experience when dealing with delivering services for patients with chronic conditions *[ask anything else – some of the above examples could be mentioned as challenges].*
  - Others: Motivation and incentives
- In general, what is being done/can be done to improve the provision of health care services for chronic conditions in this health facility

1. **COMMUNITY CHRONIC CARE NETWORK**

Let’s now talk about people or groups at the community level. Who else in the community helps/assist with supporting patients with their chronic conditions. For instance:

1. Family caregivers or guardians? What are their roles?
2. Peer patients/expert patients
   - Do you have/work with expert/peer patients in this health facility
   - What activities or forms of services do they offer to patients
   - Are these groups of value or benefit to patients? Why/why not?
3. Patient support groups

- How many support groups are there and for what types of patients?
- What activities or forms of services do they offer to members?
- Are these groups of value or benefit to patients? Why/why not?
- Any particular challenges facing these groups?

1. Community-based organizations

- Are you aware of any local CBOs that operate in this community?
- What activities or forms of services do they offer that target patients with chronic conditions?
- How does the health facility link with these groups i.e. mechanisms of interaction?
- Are these groups of value or benefit to patients? Why/why not?
- Any particular challenges facing these groups?

In general, what is being done/can be done to improve how the above groups/individuals function i.e. expert patients, CBOs, support groups?

- Finally, let’s look at other partners or organizations in this community
  - Which organizations are currently offering support to patients with chronic conditions (health and non-health)

*E.g. NGOs, Government ministries (health, social welfare, education)*

- - What activities do they do/support they provide
  - Are there actors who are important in this community who could contribute to the support and care for patients with chronic conditions;
    - who are they and what could be their potential role

1. **RECOMMENDATIONS AND CONCLUSIONS:**

- Anything else you think should be done to help improve the care and support for patient(s) with chronic condition(s)
- Any questions or comments you would like to share
