# Supplementary material for: Context matters: a qualitative study of the practicalities and dilemmas of delivering integrated chronic care within primary and secondary care settings in a rural Malawian district
Source: BMC Fam Pract. 2020 Jun 8;21:101. doi: 10.1186/s12875-020-01174-1 (PMC7282183; doi:10.1186/s12875-020-01174-1)
Supplement: Supplementary file 2 — Additional file 2. COREQ checklist. [file 12875_2020_1174_MOESM2_ESM.docx]

**Manuscript:** Context matters: A qualitative study of the practicalities and dilemmas of delivering integrated chronic care within primary and secondary care settings in a rural Malawian district.

**Consolidated criteria for reporting qualitative studies (COREQ): 32-item checklist**

Developed from:

Tong A, Sainsbury P, Craig J. Consolidated criteria for reporting qualitative research (COREQ): a 32-item checklist for interviews and focus groups. *International Journal for Quality in Health Care*. 2007. Volume 19, Number 6: pp. 349 – 357

A checklist of items that should be included in reports of qualitative research. You must report the page number in your manuscript where you consider each of the items listed in this checklist. If you have not included this information, either revise your manuscript accordingly before submitting or note N/A.

| **No. Item** | **Guide questions/description** | **Reported on Page #** |
| --- | --- | --- |
| **Domain 1: Research team and reﬂexivity** | | |
| *Personal Characteristics* | | |
| 1. Interviewer/facilitator | VA supported by a trained research assistant conducted the interviews and structured observations. | Page 8 and Acknowledgement section |
| 2. (Author) credentials | VA (Doctoral fellow), CA (PhD), JBA (Prof), BC (Prof), JVL (Prof). | Author details page |
| 3. Occupation | VA (Doctoral fellow), CA (Research Fellow), JBA (Prof), BC (Prof), JVL (Prof). | Author details page |
| 4. Gender | Three authors and the Malawian research assistant are female. Two authors are male. | Author details page |
| 5. Experience and training | VA and CA have experience in qualitative research in the sub-Saharan African region. JBA, BC and JVL have experience supervising qualitative research projects. | Author details page |
| *Relationship with participants* | | |
| 6. Relationship established | None – the research team were new to the study context. | Page 7 |
| 7. Participant knowledge of the interviewer | The interviewers and purpose of research were only made known to interviewees during consenting | Page 8 |
| 8. Interviewer characteristics | Interviews were conducted by VA, a female doctoral research fellow with extensive qualitative research experience, with support from a female Malawian research assistant trained on qualitative research methods. The interest in the research topic, which formed part of VA’s doctoral thesis were openly declared to the research participants at the point of introducing the study and obtaining informed consent. | Page 8 and 10 |
| **Domain 2: study design** | | |
| *Theoretical framework* | | |
| 9. Methodological orientation and theory | Thematic approach and applied an integrated care framework in the sub-analysis of qualitative data from a larger study on chronic care in rural Malawi | Page 6, 9 - 11 |
| *Participant selection* | | |
| 10. Sampling | Purposive sampling for participants and organizations visited identified through, snowballing approach/referrals from key informant/interviewees | Page 7 |
| 11. Method of approach | Participants were identified in consultation with senior health managers and health facility in-charges where the study was conducted | Page 7 |
| 12. Sample size | 15 interviewees and 5 representatives of the NGOs visited | Page 7 and 9 |
| 13. Non-participation | None | Page 10 |
| *Setting* | | |
| 14. Setting of data collection | At the interviewees office or workstation within heath facilities | Page 8 and 9 |
| 15. Presence of non-participants | No | Page 8 |
| 16. Description of sample | Yes and summarized in Table 1 | Page 7 and 11 |
| *Data collection* | | |
| 17. Interview guide | Topic guides included in supplementary file | Supplementary file 1 and page 9 |
| 18. Repeat interviews | No | Page 8 |
| 19. Audio/visual recording | Yes | Page 8 |
| 20. Field notes | Yes | Page 9 |
| 21. Duration | Yes | Page 8 and 9 |
| 22. Data saturation | During the sampling process, we strived to identify and interview all health cadres knowledgeable on the study topic and for representativeness of these health cadres per facility. | Page 7 – 8 |
| 23. Transcripts returned | No, findings were shared during feedback meetings | Page 10 |
| **Domain 3: analysis and ﬁndings** | | |
| *Data analysis* | | |
| 24. Number of data coders | 1 | Page 10 |
| 25. Description of the coding tree | Yes | Page 9 |
| 26. Derivation of themes | Yes | Page 9 |
| 27. Software | Yes – QSR NVivo Pro Version 11 | Page 9 |
| 28. Participant checking | Yes, a sub-sample involved during end of study feedback meetings | Page 10 |
| *Reporting* |  |  |
| 29. Quotations presented | Yes | Page 11, 15 – 21 and Supplementary file S4 |
| 30. Data and ﬁndings consistent | Yes | Page 10 – 21 |
| 31. Clarity of major themes | Yes | Page 10 – 21 |
| 32. Clarity of minor themes | Yes | Page 10 – 21 |
