# Supplementary material for: Context matters: a qualitative study of the practicalities and dilemmas of delivering integrated chronic care within primary and secondary care settings in a rural Malawian district
Source: BMC Fam Pract. 2020 Jun 8;21:101. doi: 10.1186/s12875-020-01174-1 (PMC7282183; doi:10.1186/s12875-020-01174-1)
Supplement: Supplementary file 3 — Additional file 3. Health facility structured observation guide. [file 12875_2020_1174_MOESM3_ESM.docx]

**HEALTH FACILITY OBSERVATION GUIDE**

***Location and purpose:***

***Observer(s):***

***No. of health providers present (male/female)***

***Date:***

***Start time:***

***End time:***

**CLINIC(S) VISITED**

- NCD clinic
- Palliative care
- HIV clinic
- Mental health clinic

Are there any clinics running concurrently

**GENERAL HEALTH TALK**

- Who is giving information (cadre of health personnel)
- Approximately how many patients present in waiting area (male & female)
- What information is covered by the facilitator e.g. (provide specifics of content covered)
  - Nutrition (diet modification/restrictions)
  - Medication
  - Physical exercise
  - Sexual and reproductive health
  - Clinic schedules and appointment
  - Self-management education e.g. symptom recognition, home symptom management etc.
  - Social support e.g. involving caregivers, spiritual care etc.
- How does the facilitator and patients interact:
  - Level of interaction
  - Tone of communication
  - Patient asking questions (give examples)
- Approximately how long did the health talks cover
- How many sessions of health talks are conducted and frequency?

**TRIAGE PROCESS:**

- What measurements are taken and by who. *E.g.*
  - *Weight*
  - *Height*
  - *BMI*
  - *BP measurements*
  - *Pulse reading*
  - *HIV viral load testing*
  - *Others*
- Where are these measurements taken (waiting area, in specific rooms etc.)
- How are the these measurements taken and recorded (e.g. BMI chart, digital or manual scales)
- Which measurements are not performed and why

**CLINICAL ASSESSMENT AND PATIENT-PROVIDER ENGAGEMENT**

- What is the set up in consultation room
  - Number of health worker(s) present (specify cadres if many)
  - Are the consultation sessions group-based or individual patient
  - Do patient(s) have other family members present in the consultation room
  - Check for availability of job aides/guidelines (specify e.g. posters, books or manuals)
- Clinical assessment and consultation process
  - How are patients received on arrival
  - How does the provider communicate with patients
  - Symptom reporting and diagnosis:

- Are patients encouraged to discuss health problems

- Does provider list examples of symptoms related to their condition (specify details)

- Does the provider conduct physical examination? Are explanations given for doing this

- During consultation are the providers filling in any specific forms

- Medication and monitoring

- Does provider discuss treatment plan (e.g. what medication will be given and home management instructions)

- Does provider discuss potential side effects and what to do when experience side effects

- Is the patient provided with instructions on how to take their medication

- Does the provider give alternative options (and referral places)

- In case medicines are lacking, are they provided with information on where else to find medication

- Does provider cross check with patient if they understood instructions given on treatment plan

- Does provider communicate appointment schedules with patients

**HEALTH PROMOTION AND PATIENT EDUCATION**

- Are patients provided with specific counselling about prevention strategies
- Are patients taught or informed of tell-tell signs or symptoms to observe
- Are patients informed of how to manage these symptoms while at home or first-aid skills
- Are patients given specific education for their condition e.g. foot care and wound care (diabetes)
- How are the sessions conducted i.e. in groups or as individual sessions

**HEALTH RECORDS**

- How is patient data captured (paper-based or electronic)
- Are their special forms being filled (e.g. master cards) and specify who is responsible with the task
- Is the patient explained to of the information written in their health passport

**OTHER**

Any other interesting observations or information shared
