# Supplementary material for: Context matters: a qualitative study of the practicalities and dilemmas of delivering integrated chronic care within primary and secondary care settings in a rural Malawian district
Source: BMC Fam Pract. 2020 Jun 8;21:101. doi: 10.1186/s12875-020-01174-1 (PMC7282183; doi:10.1186/s12875-020-01174-1)
Supplement: Supplementary file 4 — Additional file 4. Additional quotes. [file 12875_2020_1174_MOESM4_ESM.docx]

# Box 1: Illustrative quotes of facilitators and barriers to integrating chronic care by theme

| **Quote no.** | **Theme: Policy framework, coordination, and strengthening linkages** |
| --- | --- |
| 1 | *…when it comes to non-communicable [diseases], it is just new under the Ministry of Health here in Malawi… This [NCD] department is new but the ministry is trying to incorporate with other departments so that many people can be treated under one roof. That is why with the new HIV guidelines this time, they are talking about every person who is HIV positive, of the age 30 years and above…you need to check their blood pressure... [and] can then start treating these people. Previously, for example in the ART clinic, people were not even screened for hypertension as well as diabetes, but this time things are now changing, we are now facing another direction…because we are encompassing each and every condition so that people can be treated accordingly.* ***(KII16_Manager_District)*** |
| 2 | *…the DHO [district health office]…oversees all the activities of the district and some NGOs in the district who are also working towards improving the health services in the district like…[those] concerned with civic education. So they go to the community, they disseminate all sought of information…there are those health related NGOs…the social welfare [department] we also involve them sometimes when there are issues which need their support. We need to meet as stakeholders…because usually as a district they organize a meeting where all stakeholders meet.* ***(KII14_Manager_District)*** |
| 3 | *…we need to have review meetings. So, it is like conferencing, clinical conference on chronic case management of various type [and] several things can be unveiled, and it will be like community of practice and cross-fertilization of experiences, action points can come out, and implementing such a plan can benefit the patients. (****KII19_Manager_District)*** |
| **Theme: Organisation of chronic care services and recommendations** | |
| 4 | *…because we have static clinics… we need to cover each and everybody who is suffering from non-communicable disease as well as HIV and mental health. We have specific clinics for these people, so we try our best to observe and monitor them at each and every clinic, so I feel they are assisted…when it comes to treatment, most of the times because of erratic supply of drugs you will find that maybe sometimes we don’t have drugs.* ***(KII16_Manager_District)*** |
| 5 | *…the CBOs [community-based organisations] that have been trained well in collaboration with us [facility staff], though not common, they [CBOs] know the sick people in the villages and register them… Through the CBOs and through the chiefs, they may know that in [such a] village there is a sick person… and the chief puts them in groups so that they can receive money… the sick people or the elderly. That kind of help is happening in the villages without necessarily passing through us [facility staff] (****KII07_HSA_Public-PHC)*** |
| 6 | *…we need to be more specific in terms of communication to the community and bring services as close as possible to the community because in the [district], health facilities are far apart. We need to have vibrant outreach services, which can stick to schedule. We also must have a one-stop clinic for those who have multiple conditions and would like to have attention for both conditions, or for the conditions they have…* ***(KII19_Manager_District)*** |
| 7 | *R: We have not yet integrated these programmes like HIV and NCDs, if we have a HIV patient they come at their own date to collect drugs, and come at another date for NCD…*  *M: Having identified this as a gap has there been any opportunity maybe to ask or maybe measures towards improving integration at the district level or with partners at the national level? R: At the district level no, but with partners, they say maybe we should integrate HIV and NCD, and we should do them together. They say soon they will come, they will come in so that we assess every HIV patient, we assess NCDs, if they have any NCD condition we integrate together.* ***(KII15_Manager_District)*** |
| 8 | *…most of the NGOs they have a link towards community-based services, but the challenge which we also have is distances to nearest facilities where patients can access services, mode of travel to the facilities, and when they come to these facilities usually some services are not available on a daily basis. Sometimes it is two days in a week or one day in a week and that causes a lot of congestion on the part of the patients, and high workload on the part of the service providers.* ***(KII19_Manager_District)*** |
| 9 | *…the introduction of a special clinic day for people with chronic illnesses I think it might help. Because we could say one nurse or a clinician in a day should at least attend to that clinic, whereby those people with chronic illnesses get screened, and given medication or may be referred to other facilities to access extra care. So the introduction of this special day for the people at this facility, I think it might help to ease some of these problems.* ***(KII03_Nurse_Public-PHC)*** |
| **Theme: Health workforce capacity and supportive resources** | |
| 10 | *…sometimes we fail to reach [community-based structures] because we [facility staff] cannot move out because sometimes it would have been better if we could work with those teams in the community even with the volunteers…and see what they are doing.* ***(KII01_Nurse_Private-PHC)*** |
| 11 | *…the Ministry of Health together with partners, we have started to train people more especially when it comes to these new HIV guidelines, and I feel and I hope that each and everybody is going to be trained, so that [they] can know how to manage people with HIV…and treated according to the new guidelines. This will trickle from…central hospital, district hospital up to the health centres, each and everybody will be trained* ***(KII16_Manager_District)****.* |
| **Theme: Financing approaches and recommendations** | |
| 12 | *M: NCDs at the moment how are they funded and what is the allocation like if we compare it with other conditions? R: Of course, it has its allocation in like the essential health package, there is that component but for the monies to come out and like sponsor certain programme activities it becomes a challenge. The only thing that is happening is like providing us transport to go around for outreach clinics, the drugs here and there, but for like orientation of staff or build the capacity of staff is becoming a challenge.* ***(KII15_Manager_District)*** |
| 13 | *…partners for NCDs are the ones that we need most… I think they have to also look into this thing that whatever they might help with might not be used for people with NCDs only... We might also use for other people…what I am trying to say is whenever they are bringing their resources, whether resources in terms of supplies or people to work with us, they should also consider that resources are not meant only for their targeted people.* ***(KII03_Nurse_Public-PHC)*** |
